# Development of Human Cell-Based *In Vitro* Infection Models to Determine the Intracellular Survival of *Mycobacterium avium*

**DOI:** 10.3389/fcimb.2022.872361

**Published:** 2022-06-24

**Authors:** Gül Kilinç, Kimberley V. Walburg, Kees L. M. C. Franken, Merel L. Valkenburg, Alexandra Aubry, Mariëlle C. Haks, Anno Saris, Tom H. M. Ottenhoff

**Affiliations:** ^1^ Department of Infectious Diseases, Leiden University Medical Center, Leiden, Netherlands; ^2^ Sorbonne Université, INSERM, Centre d’Immunologie et des Maladies Infectieuses, U1135, AP-HP, Hôpital Pitié-Salpêtrière, Centre National de Référence des Mycobactéries et de la Résistance des Mycobactéries aux Antituberculeux, Paris, France

**Keywords:** *Mycobacterium avium*, primary human macrophages, infection models, drug susceptibility assays, MGIT 960 system

## Abstract

The *Mycobacterium avium* (*Mav*) complex accounts for more than 80% of all pulmonary diseases caused by non-tuberculous mycobacteria (NTM) infections, which have an alarming increase in prevalence and vary in different regions, currently reaching 0.3–9.8 per 100,000 individuals. Poor clinical outcomes, as a result of increasing microbial drug resistance and low treatment adherence due to drug-toxicities, emphasize the need for more effective treatments. Identification of more effective treatments, however, appears to be difficult, which may be due to the intracellular life of NTM and concomitant altered drug sensitivity that is not taken into account using traditional drug susceptibility testing screenings. We therefore developed human cell-based *in vitro Mav* infection models using the human MelJuSo cell line as well as primary human macrophages and a fluorescently labeled *Mav* strain. By testing a range of multiplicity of infection (MOI) and using flow cytometry and colony-forming unit (CFU) analysis, we found that an MOI of 10 was the most suitable for *Mav* infection in primary human macrophages, whereas an MOI of 50 was required to achieve similar results in MelJuSo cells. Moreover, by monitoring intracellular bacterial loads over time, the macrophages were shown to be capable of controlling the infection, while MelJuSo cells failed to do so. When comparing the MGIT system with the classical CFU counting assay to determine intracellular bacterial loads, MGIT appeared as a less labor-intensive, more precise, and more objective alternative. Next, using our macrophage *Mav* infection models, the drug efficacy of the first-line drug rifampicin and the more recently discovered bedaquiline on intracellular bacteria was compared to the activity on extracellular bacteria. The efficacy of the antibiotics inhibiting bacterial growth was significantly lower against intracellular bacteria compared to extracellular bacteria. This finding emphasizes the crucial role of the host cell during infection and drug susceptibility and highlights the usefulness of the models. Taken together, the human cell-based *Mav* infection models are reliable tools to determine the intracellular loads of *Mav*, which will enable researchers to investigate host–pathogen interactions and to evaluate the efficacy of (host-directed) therapeutic strategies against *Mav*.

## Introduction

*Mycobacterium avium* (*Mav*), a pathogen widely distributed in the environment, is a member of non-tuberculous mycobacteria (NTM). NTM infections predominantly manifest as chronic lung disease (NTM-LD), of which the prevalence has been rising over the last 30 years, being more prevalent than tuberculosis in some regions (Marras et al., 2007; Adjemian et al., 2012). The vast majority (80%) of these NTM-LD cases are caused by the *Mav* complex (Rindi and Garzelli, 2014), and the higher occurrence of *Mav*-LD is mainly observed in immunocompromised patients with structural lung conditions or immunologic and genetic disorders (Ottenhoff et al., 2002; Olivier et al., 2003; Corti and Palmero, 2008; Daley, 2017). However, despite its rarity in immunocompetent individuals (<10 cases per 100,000 people below the age of 50 years), *Mav* also causes LD without predisposing conditions, especially in elderly women (Inderlied et al., 1993; Field et al., 2004; Daley, 2017).

The treatment for *Mav* infection consists of a multidrug antibiotic regimen, including a macrolide (usually clarithromycin or azithromycin), ethambutol, and a rifamycin (rifampicin or rifabutin) (Arend et al., 2009; Daley et al., 2020), and, in severe cases, also an aminoglycoside (van Ingen et al., 2012; Kwon et al., 2019). Despite a lengthy treatment that should be maintained at least 12 months after negative sputum culture conversion, approximately 60% of treatments are unsuccessful (Xu et al., 2014). The high failure rate is largely due to drug resistance and low treatment adherence as a result of lengthiness of treatment and concomitant adverse reactions, but also because of limited treatment responses and patient relapses (Field et al., 2004; Koh et al., 2013; Kwon et al., 2019; Veziris et al., 2021). Hence, the development of new treatments to eradicate *Mav* infections is highly desired.

A promising alternative or adjunctive therapy for mycobacterial infection is host-directed therapy (HDT). HDT stimulates host cells to eliminate invading pathogens and/or counteract pathogen-induced mechanisms that prevent or impair bacterial clearance. As mycobacteria are predominantly intracellular pathogens, with many host–pathogen interactions, HDT is an appealing adjunctive therapy. By targeting infected host cells, HDT offers several advantages over antibiotics: (1) HDT has a low probability of evoking *de novo* drug resistance as the drugs do not target the pathogen; (2) HDT will most likely be effective against drug-resistant mycobacterial strains; (3) HDT could also be effective against metabolically inactive and/or non-replicating bacteria; and (4) HDT and classical antibiotic could act synergistically as both target different processes, such that antibiotic treatment duration and/or dosage (and concomitant adverse effects) might be significantly reduced. Host–pathogen interactions and HDT are extensively investigated with regard to *Mycobacterium tuberculosis* (*Mtb*), and although it is known that NTM are able to modulate host immune responses, including inhibition of phagosome maturation or host epigenetic features (Early et al., 2011; Korbee et al., 2018; Moreira et al., 2020), the limited knowledge on the host–pathogen interactions during *Mav* infections still hampers the identification of targets for HDT (Kilinc et al., 2021).

To gain further insight into host–pathogen interactions and to identify new therapeutic molecules against intracellular *Mav*, robust *in vitro* infection models in human cells are required. We previously described *in vitro* infection models for (multi-drug resistant) *Mtb* that allow accurate determination of mycobacterial loads and proved suitable to identify HDTs for *Mtb* infections (Korbee et al., 2018; Vrieling et al., 2019; Moreira et al., 2020). In the present study, we adapted and modified these models to NTM, by generating fluorescently labeled *Mav* and establishing suitable infection conditions in a human cell line as well as primary macrophages. In addition, an automated liquid culture method known as the BACTEC mycobacteria growth indicator tube (MGIT) 960 system was validated here to accurately determine intracellular bacterial loads of *Mav* (Tortoli et al., 1999). The models described here can be used to identify antimicrobial and HDT compounds and to investigate what host signaling pathways and regulatory networks control *Mav* infection.

## Materials and Methods

### Cell Cultures

The MelJuSo human melanoma cell line (kindly provided by Jacques Neefjes, Leiden University Medical Center, Leiden, the Netherlands) was maintained in Gibco Iscove’s Modified Dulbecco’s Medium (IMDM) (Life Technologies, Bleiswijk, the Netherlands) supplemented with 10% fetal bovine serum (FBS, Greiner Bio-One, Alphen a/d Rijn, the Netherlands), 100 units/ml penicillin, and 100 μg/ml streptomycin (Life Technologies) at 37°C/5% CO_2_. Peripheral blood mononuclear cells were isolated from anonymized healthy donor buffy coats obtained after written informed consent (Sanquin Blood Bank, Amsterdam, the Netherlands) by density gradient centrifugation over Ficoll Amidotrizoate (Pharmacy, LUMC, the Netherlands). This was approved by the Sanquin Ethical Advisory Board, in accordance with the Declaration of Helsinki, and according to Dutch regulations. CD14+ monocytes were isolated by magnetic cell sorting using anti-CD14-coated microbeads (Miltenyi Biotec, Bergisch Gladsbach, Germany) and differentiated for 6 days into pro-inflammatory (M1) or anti-inflammatory (M2) macrophages with 5 ng/ml of granulocyte-macrophage colony-stimulating factor (GM-CSF; Miltenyi Biotec) or 50 ng/ml macrophage-CSF (M-CSF; R&D Systems, Abingdon, UK), respectively, as previously reported (Verreck et al., 2006). Monocytes and macrophages were cultured in Gibco Dutch modified Roswell Park Memorial Institute (RPMI) 1640 medium (Life Technologies) supplemented with 10% FBS, 2 mM L-alanyl-L-glutamine (PAA, Linz, Austria), and during differentiation with 100 units/ml penicillin and 100 μg/ml streptomycin at 37°C/5% CO_2_.

### Bacterial Cultures

*Mav* laboratory strain 101 (700898, ATCC, Virginia, the United States) and three clinical isolates denoted as *Mav* 100 (amikacin-resistant), (drug-susceptible) 568, and (clarithromycin-resistant) 918 strains [the clinical isolates were isolated from pulmonary infections and displayed different susceptibility profiles to antibiotics as indicated, according to the French guidelines (Comité de l’Antibiograme de la SFM V.1.0 Avril 2021, European Committee on Antimicrobial Susceptibility Testing)] were cultured in Difco Middlebrook 7H9 broth (Becton Dickinson, Breda, the Netherlands), containing 0.2% glycerol (Merck Life Science, Amsterdam, the Netherlands), 0.05% Tween-80 (Merck Life Science), and 10% Middlebrook albumin, dextrose, and catalase (ADC) enrichment (Becton Dickinson), which was supplemented with 100 μg/ml Hygromycin B (Life Technologies) for culturing the green fluorescently labeled *Mav* Wasabi strain.

Growth of *Mav* Wasabi in suspension at 37°C was evaluated by measuring the absorbance at an optical density of 600 nm (OD_600_) using the OD_600_ Ultrospec 10 Cell density meter (Amersham Biosciences). In parallel, growth was evaluated by enumerating bacterial colonies by an agar plate assay to determine the OD factor (defined as CFU/ml in a culture with an OD_600_ of 1.0) for *Mav* Wasabi. Bacterial suspensions were therefore prepared using the estimated OD factor and plated on 7H10 square agar plates, containing Difco Middlebrook 7H10 broth (Becton Dickinson) supplemented with 10% Middlebrook oleic, albumin, dextrose and catalase (OADC) enrichment (Becton Dickinson) and 0.5% glycerol for a standard colony-forming unit (CFU) assay. Afterwards, the estimated OD factor was adjusted to the colonies counted to achieve the final OD factor. The doubling time (the time required for a population of bacteria to double in number) was calculated by first determining the doubling factor (i.e., the number of times the bacteria have doubled in numbers) by determining how many times the bacteria have doubled in numbers (c in the below equation) from early log-phase (OD_600_ = 0.25; b in the equation) until late log-phase culture (OD > 3; a in the equation).

Doubling factor = (LOG(a) − LOG(b))/LOG(c)

(As an example: Doubling factor = (LOG(3.9) − LOG(0.25))/LOG(2) = 3.96. This number indicates how many times the bacteria have doubled in numbers. When this doubling factor is corrected for the amount of time that was used, say 96 h, the doubling time of the bacteria is determined: the doubling time = time required for doubling factor/doubling factor = 3.96/96 = 24.22 h. This number indicates the time required for one generation round.

### Electroporation With and Expression of Wasabi Construct in *Mav* 101

Electroporation of *Mav* 101 was performed using the pSMT3-Wasabi construct. The Wasabi gene, amplified from the pTEC15 plasmid (Addgene plasmid #30174) by PCR, was kindly provided by Herman Spaink (Leiden University, Leiden, the Netherlands) and cloned into the mycobacterial expression vector pSMT3 (Gaora, 1998). In this vector, expression of Wasabi is constitutive and controlled by the hygromycin resistance gene-containing hsp60 promoter. First, electrocompetent *Mav* was freshly prepared from a 50-ml log-phase culture by incubation with 1.5% glycine (Life Technologies) for 18 h at 37°C. Subsequently, bacteria were centrifuged at 1,934 rcf for 20 min and washed three times with 37°C deionized H_2_O supplemented with 10% glycerol and 0.5 M sucrose (electroporation solution) followed by centrifugation at 2,120 rcf for 10 min. Electrocompetent bacteria were concentrated 100× in electroporation solution and 100 μl of bacteria was electroporated at room temperature with 5 μg of plasmid DNA using 0.2-cm-gap Gene Pulser electroporation cuvettes and the Gene Pulser Xcell Electroporation System (Bio-Rad) with the following settings: 1,000 Ω, 25 μF, 1.25 kV, and 2.5 V. Transformed bacteria were incubated overnight in 7H9 broth at 37°C in a shaking incubator, transferred to 7H10 agar plates under 100 μg/ml hygromycin selection, and incubated at 37°C/5% CO_2_ for 7–10 days.

Expression of the Wasabi green fluorescent protein in individual clones of *Mav* Wasabi was analyzed by fixating samples in Falcon Round-Bottom Polystyrene Tubes with 1% paraformaldehyde at 4°C for at least 45 min before measuring samples at wavelength 518–548 nm on the BD Accuri C6 Plus flow cytometer (BD Biosciences). FlowJo v10 Software (BD Biosciences) was used for analysis. Resistance to hygromycin was validated by mixing early log-phase *Mav* Wasabi culture with either 100 μg/ml or 200 μg/ml hygromycin, 20 μg/ml rifampicin (Sigma-Aldrich, Zwijndrecht, the Netherlands) as positive control, or DMSO (Merck Life Science) as negative control. Plates were incubated at 37°C/5% CO_2_ for 10 days. Once every 2 days, the wells were resuspended and the absorbance at 600 nm was measured using the EnVision Multimode Plate Reader (Perkin Elmer). Outgrowth of bacteria in the hygromycin condition was compared to the controls.

### *Mav* Infection of Human Cells

One day prior to infection, cultures of *Mav* Wasabi and the three clinical isolates of *Mav* were diluted to a density corresponding with early log-phase growth (OD_600_ of 0.4). On the day of infection, bacterial suspensions were diluted in appropriate cell culture medium without antibiotics to reach the indicated multiplicity of infection (MOI). MOI of the inoculum was verified by preparing tenfold serial dilutions in 7H9 medium and plating 10-μl drops of each dilution on 7H10 agar plates. For experiments using the MGIT system, 125 μl of each dilution was transferred into MGIT tubes that contain a fluorescence-quenching oxygen sensor and prepared according to the manufacturer’s protocol. Subsequently, the inoculated tubes were incubated at 37°C in a BACTEC MGIT 960 instrument and were monitored automatically for oxygen utilization, which results in an increase in fluorescence. The number of days from inoculation until cultures reached a fluorescent intensity threshold was recorded as time to positivity (TTP). The TTP measurements were plotted against plate-counted log10 CFU using linear regression to be able to calculate bacterial loads (**Supplementary Figure 1**). MelJuSo cells or primary human macrophages, seeded in flat-bottom 96-well plates at a density of 20,000 cells (2 × 10^5^ cells/ml) or 30,000 cells (3 × 10^5^ cells/ml) per well, respectively, in MelJuSo or macrophage culture medium without antibiotics 1 day before infection, were inoculated in triplicate or indicated otherwise with 100 μl of the bacterial suspension. Plates were centrifuged for 3 min at 129 rcf and incubated for 1 h at 37°C/5% CO_2_. In order to monitor only intracellular bacteria following infection, cells were washed with culture medium containing 30 μg/ml gentamicin (Merck Life Science), which blocks extracellular *Mav* growth (**Supplementary Figure 2**). Afterwards, cells were treated with fresh cell culture medium containing 5 μg/ml gentamicin and, if applicable, compounds of interest. Plates were incubated at 37°C/5% CO_2_ until readout by flow cytometry, CFU or MGIT, as indicated.

### Quantification of Infection

Cells were infected as described above, and infection rates were determined by washing cells with PBS and subsequently trypsinized with Gibco 0.05% Trypsin-EDTA (Life Technologies). After trypsinization, appropriate cell culture medium containing FBS was added to the wells to inactivate trypsin and the monolayers were scraped. Harvested cells were centrifuged in Falcon Round-Bottom Polystyrene Tubes at 453 rcf for 5 min to remove the supernatant. Cells were fixated with 1% paraformaldehyde prior to measurement and analysis as described above.

To determine numbers of bacteria taken up during infection and the subsequent survival of bacteria after prolonged incubation, infected MelJuSo cells were lysed at 0 and 24 h and primary human macrophages also at 48, 72, and 144 h post-infection using 100 μl of lysis buffer (H_2_O + 0.05% SDS). Cell lysates were serially diluted in multiple steps in 7H9 medium and 10-μl droplets were plated on 7H10 agar plates. After 7–10 days of incubation at 37°C/5% CO_2_, plates were photographically scanned, and bacterial colonies were counted. CFU counts were averaged and corrected for dilution factors to give CFU count per sample.

The ability of the MGIT system to accurately predict CFU of *Mav* was determined by evaluating intracellular bacterial loads of experimental cell lysates obtained in the same way as for the CFU analysis. Of each cell lysate, 125 μl was transferred to MGIT tubes. The obtained TTP measurements were then converted into CFU counts by using linear regression and compared with the plate-counted values. The percentage of bacterial survival was defined as the fraction of CFU measured during prolonged incubation of the total CFU measured at uptake (=100%). As part of the validation of the MGIT assay, primary human macrophages exposed to *Mav* Wasabi (10:1) were treated for 24 h with 20 μg/ml rifampicin or 0.1% DMSO as negative control. After incubation, supernatant was removed, and cells were lysed with 100 μl of lysis buffer. Number of bacteria per cell lysate was measured by both the agar plate assay and the MGIT assay. The activity of the antibiotic was determined by calculating the fraction of bacteria observed in the rifampicin condition of the total CFU measured in control (=100%).

### Application of the MGIT System to Assess the Susceptibility to Antibiotics of Intracellular Bacteria, Compared With Extracellular Bacteria

To determine the efficacy of antibiotics on extracellular bacteria, early log-phase *Mav* Wasabi culture was mixed in round-bottom 96-wells plates in duplicate with 1.29 μg/ml rifampicin, 1.74 μg/ml bedaquiline (kindly provided by Dirk Lamprecht, Janssen, Beerse, Belgium), or control (0.1% DMSO). These concentrations indicate the minimal inhibitory concentration (MIC) determined for each antibiotic by testing twofold serial drug dilutions against *Mav* Wasabi in liquid broth cultures (**Supplementary Figure 3**). Plates were incubated at 37°C/5% CO_2_ for 2 weeks. Once every 2 days, the wells were resuspended and absorbance at 600 nm was measured using the Envision Multimode Plate Reader (Perkin Elmer). For the determination of intracellular activity, primary human macrophages exposed to *Mav* Wasabi (10:1) in duplicate were treated for 24 h with 1.29 μg/ml rifampicin, 1.74 μg/ml bedaquiline, or control (0.1% DMSO). After treatment, supernatant was removed, and cells were lysed with 100 μl of lysis buffer. Cell lysates were further evaluated by the MGIT assay as described above. The activity of the antibiotics on bacteria was determined by calculating the fraction of bacteria observed in the rifampicin or bedaquiline conditions of the total CFU measured in control (=100%).

### Statistical Analysis

Normality of data was assessed using the Shapiro–Wilk test. For normally distributed paired datasets of more than two groups, we used repeated measures one-way ANOVA if data were determined by one independent variable, and repeated measures two-way ANOVA if two independent variables were involved. Paired and unpaired *t*-tests were used to evaluate differences in normally distributed datasets between two groups, whereas the Wilcoxon matched-pairs signed rank test was used for non-normally distributed paired data. To determine the strength of association between non-normally distributed datasets, the Spearman rank correlation test was used. Analyses were performed using GraphPad Prism 9.0 (GraphPad Software, San Diego, CA, USA), with *p*-values < 0.05 considered as significant.

## Results

### Generation of Fluorescently Labeled *Mav* Strain 101

**Figure 1:**
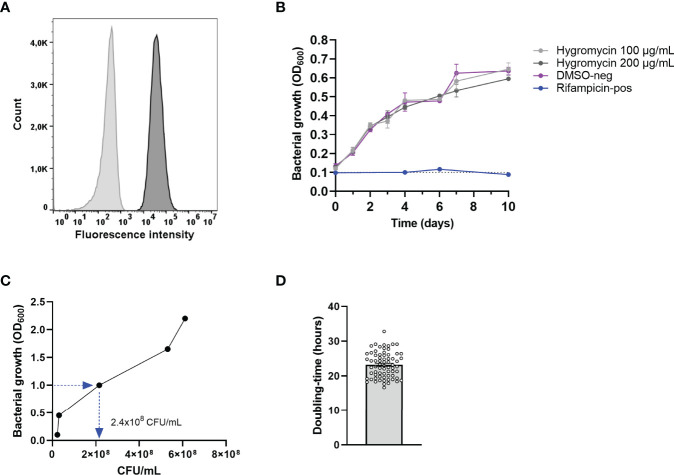
Confirmation of the generation of the green-fluorescent *Mav* Wasabi strain and its OD factor and doubling time. *Mav* was electroporated with pSMT3-Wasabi plasmid to generate a green fluorescent *Mav* strain and its fluorescence (dark gray) is presented relative to non-fluorescent *Mav* (light gray) **(A)**. *Mav* Wasabi growth in the presence of hygromycin in the indicated concentrations, DMSO (negative control), or 20 μg/ml rifampicin (positive control) was monitored by absorbance measurements at 600 nm, performed in *n* = 3 with error bars depicting SEM between experiments **(B)**. Growth kinetics of *Mav* Wasabi was monitored by measuring OD_600_ values once every 24 h, while CFU were quantified using CFU agar plate counting at the same time points. After 48 h, the bacterial density was measured to be OD_600_ of 1.0 **(C)**. The doubling time was determined as the amount of time required for the multiple generations that occurred in the *Mav* Wasabi bacterial population **(D)**. The bar and whiskers represent mean ± SEM.

The first step in developing the human cell-based *in vitro* infection models was the generation of a green fluorescent protein-expressing *Mav* strain. This was achieved by electroporating a hygromycin resistance conferring plasmid, pSMT3-Wasabi, into wild-type laboratory strain *Mav* 101. Successful transfection was confirmed by expression of the Wasabi fluorescent protein using flow cytometry (**Figure 1A**), and resistance to hygromycin by observing outgrowth (**Figure 1B**).

### Growth Kinetics of *Mav* Wasabi

The OD factor of *Mav* [the number of colony-forming units per ml (CFU/ml) in a culture with an OD_600_ value of 1.0] was determined to be able to prepare bacterial suspensions and infect cells with standardized MOI. To this end, growth kinetics of *Mav* were determined by measuring the optical density (OD_600_) and enumerating CFU of *Mav* Wasabi cultures at 0, 24, 48, 72, and 96 h after the start of the culture (**Figure 1C**). Starting in early log-phase (OD_600_ = 0.1), the bacterial culture reached an OD_600_ value of 1.0 after 48 h. At the same time point, the number of CFU/ml was obtained and verified in multiple inocula to obtain the definitive OD factor of 2.4 × 10^8^ CFU/ml.

Ultimately, the bacteria grew to an OD_600_ value of 2.2 within 96 h (**Figure 1C**). The doubling time was calculated for multiple *Mav* cultures and was determined to be 23 h on average (range: 17–33 h) (**Figure 1D**), which is in line with the slow replication rate reported in literature (Ratnatunga et al., 2020).

### *In Vitro Mav* Infection Models Using Human MelJuSo Cells and Human PBMC-Derived Primary Macrophages

**Figure 2:**
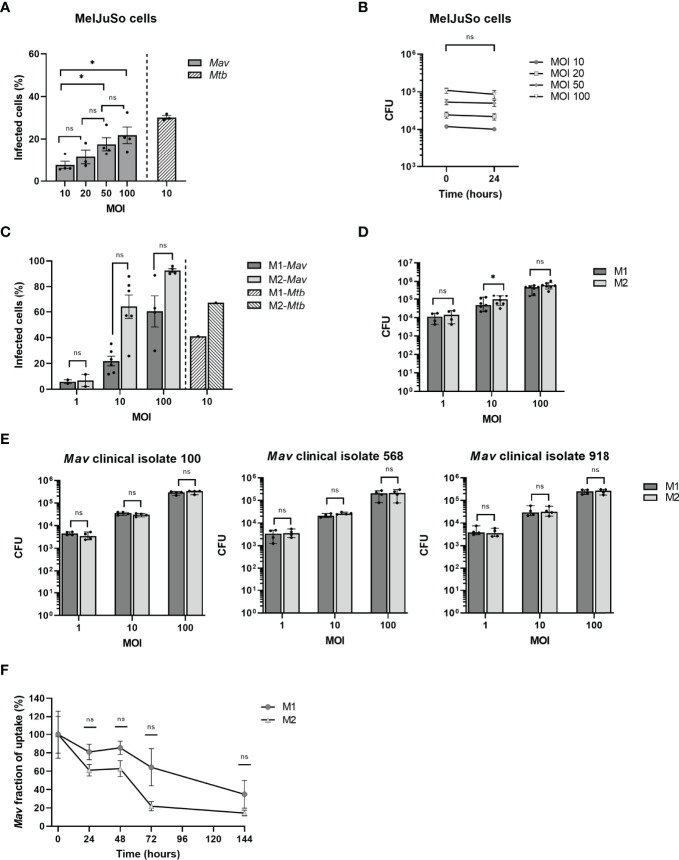
Quantification of infection with and eradication of intracellular *Mav* Wasabi and/or clinical isolates by flow cytometry and/or CFU enumeration in MelJuSo cells and primary human macrophages. MelJuSo cells were infected with a multiplicity of infection (MOI) range of *Mav* Wasabi for 1 h. Directly after infection (0 h post-infection), the percentage of infected cells was determined by flow cytometry **(A)** and intracellular bacterial load was quantified using a CFU assay **(B)**. Bacterial elimination was monitored by lysing cells for CFU analysis 24 h post-infection **(B)**. The bars and whiskers represent the mean ± SEM of four different experiments. Differences were tested for statistical significant using one-way ANOVA with Tukey’s multiple comparison testing for infection rates between indicated MOI **(A)** or two-way ANOVA with Bonferroni’s multiple comparison testing for CFU between time points for each MOI **(B)**. Monocyte-derived human macrophages differentiated into pro-inflammatory macrophages (M1) or anti-inflammatory macrophages (M2) were infected with a multiplicity of infection (MOI) range **(C, D)** or an MOI of 10 **(F)** of *Mav* Wasabi for 1 h. M1 and M2 macrophages were also exposed to an MOI range of three *Mav* clinical isolate strains 100, 568, and 918 **(E)**. Directly after infection (0 h post-infection), the percentage of infected cells was determined by flow cytometry **(C)** and intracellular bacterial load was quantified using a CFU assay **(D, E)**. In *Mav* Wasabi-infected macrophages, eradication of bacteria was monitored over time by lysing cells for CFU analysis at the indicated time points post-infection **(F)**. Primary human macrophages were obtained from 4 to 7 different donors. The bars/symbols and whiskers/error bars represent the mean ± SEM **(C, F)** or median ± range **(D, E)**. Dark and light bars represent M1 and M2, respectively. Hatched bars represent previously reported infection rates in *Mtb*-infected cells (10:1). Relevance of observed differences in infection rate and intracellular bacteria between M1 and M2 at each MOI was tested using Wilcoxon matched-pairs signed rank tests with Holm-Sidak multiple comparison testing **(C–E)**, whereas two-way ANOVA with Bonferroni’s multiple comparison testing was used for CFU between time points **(F)** *p < 0.05; ns, non-significant.

In order to investigate NTM infections at the intracellular bacterial level, we developed human cell-based infection models for *Mav*, adapted from our previously reported infection models for *Mtb* (Korbee et al., 2018; Vrieling et al., 2019). First, we evaluated the capacity of MelJuSo cells to engulf *Mav* and optimized the level of infection by adjusting the MOI to reach an infection percentage comparable to what we observed previously in our MelJuSo-*Mtb* infection model (Korbee et al., 2018). In *Mav*-infected MelJuSo cells, an MOI-dependent increase in infection was observed, as reflected by an increase in infection rate (% of infected cells) and intracellular bacterial loads directly after infection as determined by flow cytometry and CFU analysis, respectively (**Figures 2A, B**). By infecting cells for 1 h with an MOI of 10, 8% of the cells were infected as determined by flow cytometry, reflected in intracellular *Mav* counts of 1.2 × 10^4^ ± 2 × 10^3^ CFU. In contrast, *Mtb*-MelJuSo cells reached an infection rate of near 30% at an MOI of 10 (**Figure 2A**) (Korbee et al., 2018). Cells exposed to an MOI of 20, 50, or 100 of *Mav* showed a mean infection rate of 11%, 18%, or 22% and CFU counts of 2.5 × 10^4^ ± 8 × 10^3^, 5.3 × 10^4^ ± 2 × 10^4^, or 1.1 × 10^5^ ± 3 × 10^4^, respectively. After 24 h of incubation, intracellular bacterial loads were similar to bacterial loads directly after infection (**Figure 2B**), suggesting a steady state infection during the first 24 h.

In addition to the MelJuSo-*Mav* infection model, we also developed a *Mav* infection model using primary monocyte-derived human macrophages, differentiated into two diametrically opposed subsets, namely, GM-CSF-driven classically activated pro-inflammatory macrophages (M1), and M-CSF-driven alternatively activated anti-inflammatory macrophages (M2), which represent the two main phenotypes of human alveolar macrophages (Mitsi et al., 2018; Hu and Christman, 2019). A clear MOI-associated increase in infection was observed for both M1 and M2 (**Figure 2C**); using an MOI of 1, 10, and 100, M1 showed infection percentages of 6%, 22%, and 60%, respectively, while 7%, 64%, and 93% of M2 were infected. Using a similar model, the infection rates for MOI 10 *Mtb*-infected macrophages were reported to be 41% and 67% for M1 and M2, respectively (**Figure 2C**) (Korbee et al., 2018). No differences were observed in flow cytometry-based infection levels between M1 and M2, and also no consistent significant differences in numbers of CFU were observed between these cells (**Figure 2D**). In addition to the laboratory *Mav* strain, we also evaluated the phagocytosis capacity of the macrophages for the three *Mav* clinical isolates 100, 568, and 918. The uptake by M1 and M2 of these clinical isolates during infection at MOI 10 was in the same magnitude (3.3 × 10^4^ ± 5 × 10^3^, 2.4 × 10^4^ ± 4 × 10^3^ and 3.5 × 10^4^ ± 1 × 10^3^ CFU) as observed for the laboratory strain (**Figure 2E**).

The above results show that primary macrophages are more readily infected with *Mav* compared to MelJuSo cells. Using an MOI of 10 in the macrophage *Mav* model or an MOI of 50 in MelJuSo model will allow detection of at least a 3-log reduction (i.e., bacterial survival from 100% down to 0.1%), in intracellular bacterial load, which will be sufficient to identify efficacious (HDT) compounds, while at the same time not overloading the cells with bacteria.

### Primary Macrophages Are Able to Control Intracellular *Mav* Early After Infection

To determine how effective macrophages are in controlling *Mav* infection, clearance of *Mav* Wasabi by M1 and M2 exposed to MOI 10 was assessed 24, 48, 72, and 144 h post-infection (**Figure 2F**). Numbers of CFU decreased in both M1 and M2, with M2 seemingly better in controlling the infection. At the last time point, 144 h post-infection, 65 ± 20% and 86 ± 12% of intracellular bacteria were eliminated in M1 and M2, respectively (**Figure 2F**).

Additionally, we compared the intracellular elimination of *Mav* by macrophages with *Mtb* over time. We previously described kinetic analysis of intracellular *Mtb* survival in a similar M2 model, which showed a rapid reduction in *Mtb* bacterial load (Vrieling et al., 2019). These cells eliminated *Mtb* by at least 85% after 24 h, implying that *Mtb* is instantly controlled after infection, while this was less profound for *Mav* (39 ± 17%, **Figure 2F**)*. Mav* was, however, controlled to a similar extent as *Mtb* eventually (86 ± 12% and 97.8% elimination, respectively).

### MGIT as an Alternative to Quantify Intracellular Bacteria

To increase throughput and to enhance objectivity (since CFU agar plate assays are known to result in inter-observer variation when enumerating colonies), the BACTEC MGIT 960 system was used to quantify bacteria by measuring bacterial metabolic activity as a surrogate for bacterial loads.

**Figure 3:**
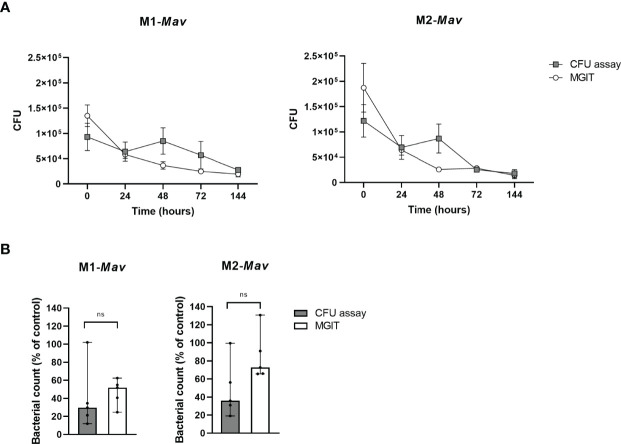
Quantification and comparison of infection with and eradication of intracellular *Mav* Wasabi by CFU enumeration based on agar plate assay and the MGIT system in primary human macrophages **(A)**. Validation of the MGIT system to determine antibiotic efficacy in primary human macrophages infected with *Mav* Wasabi **(B)**. To assess the MGIT system as a valid enumeration technique of intracellular bacteria, pro-inflammatory macrophages (M1) or anti-inflammatory macrophages (M2) were infected with an MOI 10 of *Mav* Wasabi for 1 h. After infection and during prolonged incubation, intracellular bacterial loads were quantified using the classical CFU assay and the MGIT system **(A)**. The MGIT system was validated for its use for drug testing by treating *Mav*-infected M1 and M2 (10:1) with rifampicin (20 μg/ml) or control (DMSO) for 24 h **(B)**. After treatment, cells were lysed and CFU numbers in lysates were determined by using the classical CFU assay and the MGIT assay. The symbols and whiskers represent the mean ± SEM of counted (gray boxes) and MGIT-based (open circles) CFU numbers (*n* = 3) **(A)**, whereas the bars and error bars represent the median ± range (*n* = 5) **(B)**. CFU numbers determined by either the CFU assay or MGIT were significantly correlated (Spearman *r*: 0.78; *p*-value = 0.011) **(A)** and Wilcoxon matched-pairs signed rank tests with Holm-Sidak multiple comparison testing was used to compare compound-induced effects between both methods **(B)**. Ns, non-significant.

Intracellular bacterial loads of *Mav*-infected macrophages estimated by the MGIT significantly correlated with the CFU counted from plates (Spearman *r*: 0.78; *p*-value = 0.011) and intra-assay variation for data obtained with the MGIT seemed to be smaller (coefficient of variation: 36% compared to 51% for plate-counted CFU analysis; *p*-value = 0.109) (**Figure 3A**).

To obtain further insight into the usefulness of our infection model, we compared the MGIT system to determine the activity of first-line antibiotic rifampicin on intracellular *Mav* to the classical CFU assay (**Figure 3B**). Rifampicin-induced effects determined by MGIT are in concordance with the classical CFU assay for both M1 and M2. This indicates that the MGIT system, which showed a trend of higher CFU numbers possibly due to the liquid medium as an inherent characteristic, was able to observe a compound-induced effect. Additionally, the intra-assay variation in MGIT seemed to be smaller compared to the classical CFU assay (coefficient of variation: 32% versus 78%, respectively; *p*-value = 0.170), as observed in **Figure 3A**. Based on these data, we considered the MGIT system as a viable alternative to plate-counting CFU analysis for the determination of intracellular bacterial loads.

Currently, the gold standard to evaluate antibacterial activity of chemical compounds is by monitoring the growth of bacteria in the extracellular space (i.e., broth microdilutions) (Brown-Elliott et al., 2012). Also identified in this way was the first new tuberculosis drug in several decades, bedaquiline, which showed bactericidal activity against (multi-drug resistant) *Mtb* but has also shown promising results against extracellular *Mav* and other NTM *in vitro* (Mahajan, 2013; Aguilar-Ayala et al., 2017; Brown-Elliott et al., 2017; Vesenbeckh et al., 2017). Interestingly, cases of bedaquiline resistance have also been reported (Philley et al., 2015; Alexander et al., 2017; Veziris et al., 2017). Here, we applied the MGIT system to drug susceptibility testing (DST) by determining the susceptibility to both rifampicin and bedaquiline of intracellular *Mav* (within M1) in comparison to extracellular bacteria (in liquid broth).

**Figure 4:**
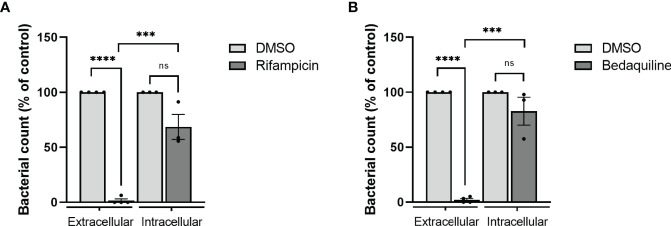
Evaluation of drug susceptibility of *Mav* Wasabi extracellularly in liquid broth versus intracellularly in primary human macrophages. To determine differential susceptibility of intracellular versus extracellular *Mav* to antibiotics, *Mav* Wasabi in liquid broth was cultured with a range of concentrations of rifampicin **(A)**, bedaquiline **(B)**, or control (DMSO). Bacterial outgrowth was monitored by absorbance measurements at 600 nm. After 14 days of incubation, the minimum concentration in which rifampicin **(A)** and bedaquiline **(B)** was assessed was 1.29 μg/ml and 1.74 μg/ml, respectively, and was used for intracellular activity evaluation. Pro-inflammatory macrophages (M1) were infected with an MOI 10 of *Mav* Wasabi for 1 h. After infection, cells were treated with rifampicin (1.29 μg/ml), bedaquiline (1.74 μg/ml), or control (DMSO) for 24 h. After treatment, cells were lysed, and intracellular bacterial loads were determined by the MGIT system. The bar and whiskers represent the mean ± SEM of extracellular (*n* = 4) or intracellular (*n* = 3) experiments. Statistics were performed using paired *t*-tests to compare the activity of antibiotic to control within each type of experiment, and unpaired *t*-tests were used to determine differences between potency of antibiotic against extracellular versus intracellular bacteria. ***p < 0.001, ****p < 0.0001; ns, non-significant.

While a concentration of 1.29 μg/ml rifampicin significantly impaired growth of extracellular bacteria (97% as compared to untreated controls), only a 31% reduction was observed in intracellular bacteria (**Figure 4A**). In line, bedaquiline treatment (1.74 μg/ml) impaired extracellular bacterial growth completely, while intracellular bacteria were only reduced by 17% as compared to untreated controls (**Figure 4B**). These findings show the higher susceptibility of extracellular bacteria to antibiotics, indicating that extracellular drug testing might overestimate bacterial susceptibility to treatments during the course of intracellular infection *in vivo*. Taken together, our *Mav* macrophage model facilitates screening of antibacterial agents against intracellular *Mav* and emphasizes the importance of measuring the intracellular compartment on antibiotic susceptibility.

## Discussion

The incidence of *Mav* pulmonary disease is increasing rapidly (Griffith et al., 2007; Kendall and Winthrop, 2013), whose therapy, despite being long and comprising multiple drugs, still has poor efficacy, as illustrated by the estimated poor cure rate of about 39% (Xu et al., 2014). The limited treatment success may be due to the fact that development of new drugs is routinely tested using DST (Griffith et al., 2007), that is, on extracellular bacteria, while *Mav* is an intracellular pathogen whose drug sensitivity may be vastly different intracellularly as compared to extracellularly. We therefore aimed to set up a model to determine the intracellular numbers of *Mav* and the two present models, one using a human phagocytic (melanoma derived) cell line and one with primary human macrophages. In these models, the viability of intracellular bacteria could be monitored and quantified over time using a classical CFU assay as well as the MGIT assay. Our models identified that the activity of the first-line drug rifampicin and the new class antibiotic bedaquiline was 3.1-fold and 5.7-fold less potent on intracellular bacteria as compared to extracellular bacteria, which may be caused by altered bacterial biology within host cells that affects drug susceptibility and/or limited exposure to antibiotics. The latter is at least partially involved as intracellular drug concentrations of rifampicin and bedaquiline have been shown to be lower than drug treatment concentrations (Kumar et al., 2006; Tanner et al., 2021). Hence, our findings emphasize the importance of taking the intracellular efficacy of an antibiotic regimen into account, for which the models presented can be exploited.

Macrophages are known to play an essential role in *Mav* infections and many host–pathogen interactions occur, of which the exact mechanisms remain to be elucidated (de Chastellier and Thilo, 2002; McGarvey and Bermudez, 2002; Rocco and Irani, 2011; Kilinc et al., 2021). To decipher these mechanisms in the natural niche of *Mav*, we developed a model that uses primary human monocyte-derived macrophages that can be used to study infections up to at least 6 days post infection. Although using primary cells is physiologically more relevant, limits on numbers of available cells and particularly inter-donor variation restrict its use in high- and medium-throughput screenings. In literature, models using cell lines THP-1 and U937 (Orme et al., 1994; Garcia et al., 2000; Danelishvili et al., 2004; Ichimura et al., 2016) have been used. These, however, require PMA stimulation, which largely disrupts and/or interferes with intracellular signaling pathways and is thereby unsuitable to identify novel HDTs (Liu and Heckman, 1998; Wu-Zhang and Newton, 2013). To circumvent this limitation, we have adapted a model using MelJuSo cells, which we have previously used to study *Mtb* infections and which do not require such pre-stimulation (Korbee et al., 2018). The MelJuSo cell line is derived from human melanocytes, and the latter have been shown to share several important characteristics with professional phagocytes like macrophages: (1) melanocytes have acidic and hydrolyse-containing vesicles, melanosomes, which very likely can function as lysosomes present in primary macrophages (Le Poole et al., 1993b); (2) melanocytes can also produce superoxides, which are one of the important antibacterial molecules produced by macrophages; and (3) human melanocytes also have been shown to process and present mycobacterial antigens to human T cells (Le Poole et al., 1993a; van Ham et al., 1997; van Ham et al., 2000). The functional immune characteristics shared between melanocytes and macrophages are indirectly supported by Korbee et al. (2018), who showed that the activity of published as well as newly discovered host-directed compounds in MelJuSo cells could be validated in human macrophages. Thus, whereas the MelJuSo model allows medium-throughput HDT compound screenings, relevant hits can be validated in the low-throughput primary macrophage model.

During mycobacterial infections, many host–pathogen interactions are at play that modulate both innate and adaptive immune responses to a large extent and are exploited by mycobacteria to facilitate bacterial survival. Consequently, modulating these interactions in favor of the host using so-called HDTs are appealing to improve the outcome. The presented model system is most suitable to study HDTs that target intracellular processes within macrophages, but cannot assess the effects of HDTs acting systemically, including promoting adaptive immune responses. However, the impact of HDTs on macrophage-mediated antigen presentation can be assessed in our new model. While, for *Mtb*, many potent effector functions of macrophages have been shown to be manipulated as part of *Mtb*’s strategy to survive intracellularly, our understanding of host–pathogen interactions of *Mav* is limited (Rocco and Irani, 2011; Upadhyay et al., 2018; Abreu et al., 2020; Kilinc et al., 2021). To improve our understanding of these processes, the models presented in this paper are ideally suitable and can furthermore be exploited to identify HDTs to improve treatment of *Mav*.

Quantification of mycobacteria is traditionally done using CFU assays, despite being labor-intensive, time-consuming, and prone to inter-individual variation. To improve objectivity and robustness, we validated the BACTEC MGIT 960 system, a liquid culture system with fully automated detection to monitor intracellular bacteria over time, by showing a strong correlation with the CFU assay, but with seemingly less variation. The MGIT has already been shown to be a robust, objective, and valid system for direct and indirect DST against *Mtb* (Huang et al., 2004; Shin et al., 2007; Jhamb et al., 2014; Kolibab et al., 2014), which is in line with the previously identified concordance between MGIT measurements and CFU counting on solid media (Pheiffer et al., 2008; Diacon et al., 2010). The MGIT system, however, measures metabolic activity in a liquid culture while CFU assays rely on growth on solid media, which might be differently affected by certain treatments. It has been shown that liquid medium offers a higher mycobacterial recovery rate, likely due to a wider range of mycobacterial populations being able to outgrow in liquid, but not in solid cultures, and liquid broth thereby enables growth of mycobacterial populations, which can also be present *in vivo* (Dhillon et al., 2004; Mitchison and Coates, 2004). In line with this, rifampicin treatment appeared to be more effective in the conventional CFU assay, as compared to MGIT, which likely is merely a reflection of bacterial colonies that are unable to grow on solid agar after rifampicin treatment than being a real effect. Consequently, enumeration of CFU on solid media could underestimate the residual mycobacterial populations after anti-*Mav* treatment, and MGIT may be a better indicator of mycobacterial survival and, therefore, physiologically more relevant.

Here, by establishing the optimal infection conditions, we developed *in vitro* human cell-based infection models for *Mav*. Both the MelJuSo cell line and primary human macrophages were capable of phagocytosing *Mav*, and intracellular survival of *Mav* within primary macrophages could be evaluated by using the MGIT system as an alternative to the classical CFU assay. The relevance and importance of such *Mav* infection models is highlighted by our finding that antibiotics were unable to eradicate intracellular *Mav*, while extracellular bacteria exposed to the same drug concentration were eliminated. Taken together, the models described here can be used to improve *Mav* therapy by also taking into account intracellular bacteria, and furthermore to advance our understanding of host–pathogen interactions and ultimately develop (host-directed) therapies to combat *Mav* infections.

## Data Availability Statement

The raw data supporting the conclusions of this article will be made available by the authors, without undue reservation.

## Author Contributions

GK designed and performed the experiments, analyzed the data, and drafted the figures. Construction of the pSMT3 construct was done by KF, whereas KW performed the electroporation. MV contributed to performing experiments. GK, MH, TO, and AS contributed to the interpretation of the results. AA isolated and provided the clinical isolates. GK wrote the manuscript. MH, AS, and TO supervised the project and, together with AA, provided critical revision of the manuscript. All authors contributed to the article and approved the submitted version.

## Funding

This project has received funding from the Innovative Medicines Initiative 2 Joint Undertaking (IMI2 JU) (www.imi.europa.eu) under the RespiriNTM (grant N° 853932) project within the IMI AntiMicrobial Resistance (AMR) Accelerator program. The JU receives support from the European Union’s Horizon 2020 research and innovation programme and EFPIA. The funders had no role in study design, data collection and analysis, decision to publish, or preparation of the manuscript. All claims expressed in this article reflect solely the authors’ view and do not necessarily represent those of the JU. The JU is not responsible for any use that may be made of the information it contains.

## Conflict of Interest

The authors declare that the research was conducted in the absence of any commercial or financial relationships that could be construed as a potential conflict of interest.

## Publisher’s Note

All claims expressed in this article are solely those of the authors and do not necessarily represent those of their affiliated organizations, or those of the publisher, the editors and the reviewers. Any product that may be evaluated in this article, or claim that may be made by its manufacturer, is not guaranteed or endorsed by the publisher.

## References

[B1] Abreu R. Giri P. Quinn F. (2020). Host-Pathogen Interaction as a Novel Target for Host-Directed Therapies in Tuberculosis. Front. Immunol. 11. doi: 10.3389/fimmu.2020.01553 PMC7396704 32849525

[B2] Adjemian J. Olivier K. N. Seitz A. E. Holland S. M. Prevots D. R. (2012). Prevalence of Nontuberculous Mycobacterial Lung Disease in U.S. Medicare Beneficiaries. Am. J. Respir. Crit. Care Med. 185 (8), 881–886. doi: 10.1164/rccm.201111-2016OC 22312016 PMC3360574

[B3] Aguilar-Ayala D. A. Cnockaert M. Andre E. Andries K. Gonzalez Y. M. J. A. Vandamme P. . (2017). *In Vitro* Activity of Bedaquiline Against Rapidly Growing Nontuberculous Mycobacteria. J. Med. Microbiol. 66 (8), 1140–1143. doi: 10.1099/jmm.0.000537 28749330 PMC5817190

[B4] Alexander D. C. Vasireddy R. Vasireddy S. Philley J. V. Brown-Elliott B. A. Perry B. J. . (2017). Emergence of Mmpt5 Variants During Bedaquiline Treatment of Mycobacterium Intracellulare Lung Disease. J. Clin. Microbiol. 55 (2), 574–584. doi: 10.1128/JCM.02087-16 27927925 PMC5277528

[B5] Arend S. M. van Soolingen D. Ottenhoff T. H. (2009). Diagnosis and Treatment of Lung Infection With Nontuberculous Mycobacteria. Curr. Opin. Pulm. Med. 15 (3), 201–208. doi: 10.1097/MCP.0b013e3283292679 19305349

[B6] Brown-Elliott B. A. Nash K. A. Wallace R. J. Jr. (2012). Antimicrobial Susceptibility Testing, Drug Resistance Mechanisms, and Therapy of Infections With Nontuberculous Mycobacteria. Clin. Microbiol. Rev. 25 (3), 545–582. doi: 10.1128/CMR.05030-11 22763637 PMC3416486

[B7] Brown-Elliott B. A. Philley J. V. Griffith D. E. Thakkar F. Wallace R. J. Jr. (2017). In Vitro Susceptibility Testing of Bedaquiline Against Mycobacterium Avium Complex. Antimicrob. Agents Chemother. 61 (2), e01798–16. doi: 10.1128/AAC.01798-16 27872065 PMC5278735

[B8] Corti M. Palmero D. (2008). Mycobacterium Avium Complex Infection in HIV/AIDS Patients. Expert Rev. Anti-Infect. Ther. 6 (3), 351–363. doi: 10.1586/14787210.6.3.351 18588499

[B9] Daley C. L. (2017). Mycobacterium Avium Complex Disease. Microbiol. Spectr. 5 (2), e1-e36. doi: 10.1128/microbiolspec.TNMI7-0045-2017 PMC11687487 28429679

[B10] Daley C. L. Iaccarino J. M. Lange C. Cambau E. Wallace R. J. Jr. Andrejak C. . (2020). Treatment of Nontuberculous Mycobacterial Pulmonary Disease: An Official ATS/ERS/ESCMID/IDSA Clinical Practice Guideline. Eur. Respir. J. 71 (4), e1–e36. doi: 10.1183/13993003.00535-2020 PMC7768748 32628747

[B11] Danelishvili L. Poort M. J. Bermudez L. E. (2004). Identification of Mycobacterium Avium Genes Up-Regulated in Cultured Macrophages and in Mice. FEMS Microbiol. Lett. 239 (1), 41–49. doi: 10.1016/j.femsle.2004.08.014 15451099

[B12] de Chastellier C. Thilo L. (2002). Pathogenic Mycobacterium Avium Remodels the Phagosome Membrane in Macrophages Within Days After Infection. Eur. J. Cell Biol. 81 (1), 17–25. doi: 10.1078/0171-9335-00220 11893075

[B13] Dhillon J. Lowrie D. B. Mitchison D. A. (2004). Mycobacterium Tuberculosis From Chronic Murine Infections That Grows in Liquid But Not on Solid Medium. BMC Infect. Dis. 4, 51. doi: 10.1186/1471-2334-4-51 15548322 PMC534102

[B14] Diacon A. H. Maritz J. S. Venter A. van Helden P. D. Andries K. McNeeley D. F. . (2010). Time to Detection of the Growth of Mycobacterium Tuberculosis in MGIT 960 for Determining the Early Bactericidal Activity of Antituberculosis Agents. Eur. J. Clin. Microbiol. Infect. Dis. 29 (12), 1561–1565. doi: 10.1007/s10096-010-1043-7 20820832

[B15] Early J. Fischer K. Bermudez L. E. (2011). Mycobacterium Avium Uses Apoptotic Macrophages as Tools for Spreading. Microb. Pathogen. 50 (2), 132–139. doi: 10.1016/j.micpath.2010.12.004 21167273 PMC3030681

[B16] Field S. K. Fisher D. Cowie R. L. (2004). Mycobacterium Avium Complex Pulmonary Disease in Patients Without HIV Infection. Chest 126 (2), 566–581. doi: 10.1378/chest.126.2.566 15302746

[B17] Gaora P. O. (1998). Expression of Genes in Mycobacteria. Methods Mol. Biol. 101, 261–273. doi: 10.1385/0-89603-471-2:261 9921485

[B18] Garcia R. C. Banfi E. Pittis M. G. (2000). Infection of Macrophage-Like THP-1 Cells With Mycobacterium Avium Results in a Decrease in Their Ability to Phosphorylate Nucleolin. Infect. Immun. 68 (6), 3121–3128. doi: 10.1128/IAI.68.6.3121-3128.2000 10816453 PMC97542

[B19] Griffith D. E. Aksamit T. Brown-Elliott B. A. Catanzaro A. Daley C. Gordin F. . (2007). An Official ATS/IDSA Statement: Diagnosis, Treatment, and Prevention of Nontuberculous Mycobacterial Diseases. Am. J. Respir. Crit. Care Med. 175 (4), 367–416. doi: 10.1164/rccm.200604-571ST 17277290

[B20] Huang T. S. Lee S. S. J. Tu H. Z. Huang W. K. Chen Y. S. Huang C. K. . (2004). Use of MGIT 960 for Rapid Quantitative Measurement of the Susceptibility of Mycobacterium Tuberculosis Complex to Ciprofloxacin and Ethionamide. J. Antimicrob. Chemother. 53 (4), 600–603. doi: 10.1093/jac/dkh120 14973155

[B21] Hu G. Christman J. W. (2019). Editorial: Alveolar Macrophages in Lung Inflammation and Resolution. Front. Immunol. 10 2275. doi: 10.3389/fimmu.2019.02275 31616438 PMC6768960

[B22] Ichimura N. Sato M. Yoshimoto A. Yano K. Ohkawa R. Kasama T. . (2016). High-Density Lipoprotein Binds to Mycobacterium Avium and Affects the Infection of THP-1 Macrophages. J. Lipids 2016, 4353620. doi: 10.1155/2016/4353620 27516907 PMC4969507

[B23] Inderlied C. B. Kemper C. A. Bermudez L. E. (1993). The Mycobacterium Avium Complex. Clin. Microbiol. Rev. 6 (3), 266–310. doi: 10.1128/cmr.6.3.266 8358707 PMC358286

[B24] Jhamb S. S. Goyal A. Singh P. P. (2014). Determination of the Activity of Standard Anti-Tuberculosis Drugs Against Intramacrophage Mycobacterium Tuberculosis, *In Vitro*: MGIT 960 as a Viable Alternative for BACTEC 460. Braz. J. Infect. Dis. 18 (3), 336–340. doi: 10.1016/j.bjid.2013.12.004 24709416 PMC9427504

[B25] Kendall B. A. Winthrop K. L. (2013). Update on the Epidemiology of Pulmonary Nontuberculous Mycobacterial Infections Preface. Semin. Respir. Crit. Care Med. 34 (1), 87–94. doi: 10.1055/s-0033-1333567 23460008

[B26] Kilinc G. Saris A. Ottenhoff T. H. M. Haks M. C. (2021). Host-Directed Therapy to Combat Mycobacterial Infections. Immunol. Rev. 301 (1), 62–83. doi: 10.1111/imr.12951 33565103 PMC8248113

[B27] Koh W. J. Hong G. Kim S. Y. Jeong B. H. Park H. Y. Jeon K. . (2013). Treatment of Refractory Mycobacterium Avium Complex Lung Disease With a Moxifloxacin-Containing Regimen. Antimicrob. Agents Chemother. 57 (5), 2281–2285. doi: 10.1128/Aac.02281-12 23478956 PMC3632919

[B28] Kolibab K. Yang A. Parra M. Derrick S. C. Morris S. L. (2014). Time to Detection of Mycobacterium Tuberculosis Using the MGIT 320 System Correlates With Colony Counting in Preclinical Testing of New Vaccines. Clin. Vaccine Immunol. 21 (3), 453–455. doi: 10.1128/Cvi.00742-13 24371256 PMC3957671

[B29] Korbee C. J. Heemskerk M. T. Kocev D. van Strijen E. Rabiee O. Franken K. . (2018). Combined Chemical Genetics and Data-Driven Bioinformatics Approach Identifies Receptor Tyrosine Kinase Inhibitors as Host-Directed Antimicrobials. Nat. Commun. 9 (1), 358. doi: 10.1038/s41467-017-02777-6 29367740 PMC5783939

[B30] Kumar P. V. Asthana A. Dutta T. Jain N. K. (2006). Intracellular Macrophage Uptake of Rifampicin Loaded Mannosylated Dendrimers. J. Drug Target. 14 (8), 546–556. doi: 10.1080/10611860600825159 17050121

[B31] Kwon Y. S. Koh W. J. Daley C. L. (2019). Treatment of Mycobacterium Avium Complex Pulmonary Disease. Tuberc. Respir. Dis. (Seoul). 82 (1), 15–26. doi: 10.4046/trd.2018.0060 30574687 PMC6304322

[B32] Le Poole I. C. Mutis T. van den Wijngaard R. M. Westerhof W. Ottenhoff T. de Vries R. R. . (1993a). A Novel, Antigen-Presenting Function of Melanocytes and its Possible Relationship to Hypopigmentary Disorders. J. Immunol. 151 (12), 7284–7292. 8258725

[B33] Le Poole I. C. van den Wijngaard R. M. Westerhof W. Verkruisen R. P. Dutrieux R. P. Dingemans K. P. . (1993b). Phagocytosis by Normal Human Melanocytes *In Vitro* . Exp. Cell Res. 205 (2), 388–395. doi: 10.1006/excr.1993.1102 8482344

[B34] Liu W. S. Heckman C. A. (1998). The Sevenfold Way of PKC Regulation. Cell Signal 10 (8), 529–542. doi: 10.1016/s0898-6568(98)00012-6 9794251

[B35] Mahajan R. (2013). Bedaquiline: First FDA-Approved Tuberculosis Drug in 40 Years. Int. J. Appl. Basic. Med. Res. 3 (1), 1–2. doi: 10.4103/2229-516X.112228 23776831 PMC3678673

[B36] Marras T. K. Chedore P. Ying A. M. Jamieson F. (2007). Isolation Prevalence of Pulmonary non-Tuberculous Mycobacteria in Ontario 1997 2003. Thorax 62 (8), 661–666. doi: 10.1136/thx.2006.070797 17311842 PMC2117272

[B37] McGarvey J. Bermudez L. E. (2002). Pathogenesis of Nontuberculous Mycobacteria Infections. Clin. Chest. Med. 23 (3), 569–583. doi: 10.1016/s0272-5231(02)00012-6 12370993

[B38] Mitchison D. A. Coates A. R. (2004). Predictive *In Vitro* Models of the Sterilizing Activity of Anti-Tuberculosis Drugs. Curr. Pharm. Des. 10 (26), 3285–3295. doi: 10.2174/1381612043383269 15544516

[B39] Mitsi E. Kamng'ona R. Rylance J. Solorzano C. Jesus Reine J. Mwandumba H. C. . (2018). Human Alveolar Macrophages Predominately Express Combined Classical M1 and M2 Surface Markers in Steady State. Respir. Res. 19 (1), 66. doi: 10.1186/s12931-018-0777-0 29669565 PMC5907303

[B40] Moreira J. D. Koch B. E. V. van Veen S. Walburg K. V. Vrieling F. Guimaraes M. P. D. . (2020). Functional Inhibition of Host Histone Deacetylases (HDACs) Enhances *In Vitro* and *In Vivo* Anti-Mycobacterial Activity in Human Macrophages and in Zebrafish. Front. Immunol. 11. doi: 10.3389/fimmu.2020.00036 PMC7008710 32117228

[B41] Olivier K. N. Weber D. J. Wallace R. J. Jr. Faiz A. R. Lee J. H. Zhang Y. . (2003). Nontuberculous Mycobacteria. I: Multicenter Prevalence Study in Cystic Fibrosis. Am. J. Respir. Crit. Care Med. 167 (6), 828–834. doi: 10.1164/rccm.200207-678OC 12433668

[B42] Orme I. M. Roberts A. D. Furney S. K. Skinner P. S. (1994). Animal and Cell-Culture Models for the Study of Mycobacterial Infections and Treatment. Eur. J. Clin. Microbiol. Infect. Dis. 13 (11), 994–999. doi: 10.1007/Bf02111500 7698125

[B43] Ottenhoff T. H. Verreck F. A. Lichtenauer-Kaligis E. G. Hoeve M. A. Sanal O. van Dissel J. T. (2002). Genetics, Cytokines and Human Infectious Disease: Lessons From Weakly Pathogenic Mycobacteria and Salmonellae. Nat. Genet. 32 (1), 97–105. doi: 10.1038/ng0902-97 12205477

[B44] Pheiffer C. Carroll N. M. Beyers N. Donald P. Duncan K. Uys P. . (2008). Time to Detection of Mycobacterium Tuberculosis in BACTEC Systems as a Viable Alternative to Colony Counting. Int. J. Tuberculosis. Lung Dis. 12 (7), 792–798. 18544206

[B45] Philley J. V. Wallace R. J. Benwill J. L. Taskar V. Brown-Elliott B. A. Thakkar F. . (2015). Preliminary Results of Bedaquiline as Salvage Therapy for Patients With Nontuberculous Mycobacterial Lung Disease. Chest 148 (2), 499–506. doi: 10.1378/chest.14-2764 25675393 PMC4694173

[B46] Ratnatunga C. N. Lutzky V. P. Kupz A. Doolan D. L. Reid D. W. Field M. . (2020). The Rise of Non-Tuberculosis Mycobacterial Lung Disease. Front. Immunol. 11. doi: 10.3389/fimmu.2020.00303 PMC7062685 32194556

[B47] Rindi L. Garzelli C. (2014). Genetic Diversity and Phylogeny of Mycobacterium Avium. Infect. Genet. Evol. 21, 375–383. doi: 10.1016/j.meegid.2013.12.007 24345519

[B48] Rocco J. M. Irani V. R. (2011). Mycobacterium Avium and Modulation of the Host Macrophage Immune Mechanisms. Int. J. Tuberc. Lung Dis. 15 (4), 447–452. doi: 10.5588/ijtld.09.0695 21396201

[B49] Shin S. J. Han J. H. Manning E. J. B. Collins M. T. (2007). Rapid and Reliable Method for Quantification of Mycobacterium Paratuberculosis by Use of the BACTEC MGIT 960 System. J. Clin. Microbiol. 45 (6), 1941–1948. doi: 10.1128/Jcm.02616-06 17428943 PMC1933085

[B50] Tanner L. Mashabela G. T. Omollo C. C. de Wet T. J. Parkinson C. J. Warner D. F. . (2021). Intracellular Accumulation of Novel and Clinically Used TB Drugs Potentiates Intracellular Synergy. Microbiol. Spectr. 9 (2), e0043421. doi: 10.1128/Spectrum.00434-21 34585951 PMC8557888

[B51] Tortoli E. Cichero P. Piersimoni C. Simonetti M. T. Gesu G. Nista D. (1999). Use of BACTEC MGIT 960 for Recovery of Mycobacteria From Clinical Specimens: Multicenter Study. J. Clin. Microbiol. 37 (11), 3578–3582. doi: 10.1128/JCM.37.11.3578-3582.1999 10523555 PMC85696

[B52] Upadhyay S. Mittal E. Philips J. (2018). Tuberculosis and the Art of Macrophage Manipulation. Pathog. Dis. 76 (4), fty037. doi: 10.1093/femspd/fty037 PMC6251593 29762680

[B53] van Ham S. M. Tjin E. P. Lillemeier B. F. Gruneberg U. van Meijgaarden K. E. Pastoors L. . (1997). HLA-DO is a Negative Modulator of HLA-DM-Mediated MHC Class II Peptide Loading. Curr. Biol. 7 (12), 950–957. doi: 10.1016/s0960-9822(06)00414-3 9382849

[B54] van Ham M. van Lith M. Lillemeier B. Tjin E. Gruneberg U. Rahman D. . (2000). Modulation of the Major Histocompatibility Complex Class II-Associated Peptide Repertoire by Human Histocompatibility Leukocyte Antigen (HLA)-Do. J. Exp. Med. 191 (7), 1127–1135. doi: 10.1084/jem.191.7.1127 10748231 PMC2193174

[B55] van Ingen J. Boeree M. J. van Soolingen D. Mouton J. W. (2012). Resistance Mechanisms and Drug Susceptibility Testing of Nontuberculous Mycobacteria. Drug Resist. Update 15 (3), 149–161. doi: 10.1016/j.drup.2012.04.001 22525524

[B56] Verreck F. A. de Boer T. Langenberg D. M. van der Zanden L. Ottenhoff T. H. (2006). Phenotypic and Functional Profiling of Human Proinflammatory Type-1 and Anti-Inflammatory Type-2 Macrophages in Response to Microbial Antigens and IFN-Gamma- and CD40L-Mediated Costimulation. J. Leukoc. Biol. 79 (2), 285–293. doi: 10.1189/jlb.0105015 16330536

[B57] Vesenbeckh S. Schonfeld N. Roth A. Bettermann G. Krieger D. Bauer T. T. . (2017). Bedaquiline as a Potential Agent in the Treatment of Mycobacterium Abscessus Infections. Eur. Respir. J. 49 (5), 1700083. doi: 10.1183/13993003.00083-2017 28529203

[B58] Veziris N. Andrejak C. Bouee S. Emery C. Obradovic M. Chiron R. (2021). Non-Tuberculous Mycobacterial Pulmonary Diseases in France: An 8 Years Nationwide Study. BMC Infect. Dis. 21 (1), 1165 doi: 10.1186/s12879-021-06825-x 34789152 PMC8600813

[B59] Veziris N. Bernard C. Guglielmetti L. Le Du D. Marigot-Outtandy D. Jaspard M. . (2017). Rapid Emergence of Mycobacterium Tuberculosis Bedaquiline Resistance: Lessons to Avoid Repeating Past Errors. Eur. Respir. J. 49 (3), 1601719. doi: 10.1183/13993003.01719-2016 28182568

[B60] Vrieling F. Wilson L. Rensen P. C. N. Walzl G. Ottenhoff T. H. M. Joosten S. A. (2019). Oxidized Low-Density Lipoprotein (oxLDL) Supports Mycobacterium Tuberculosis Survival in Macrophages by Inducing Lysosomal Dysfunction. PLoS Pathog. 15 (4), e1007724. doi: 10.1371/journal.ppat.1007724 30998773 PMC6490946

[B61] Wu-Zhang A. X. Newton A. C. (2013). Protein Kinase C Pharmacology: Refining the Toolbox. Biochem. J. 452 (2), 195–209. doi: 10.1042/BJ20130220 23662807 PMC4079666

[B62] Xu H. B. Jiang R. H. Li L. (2014). Treatment Outcomes for Mycobacterium Avium Complex: A Systematic Review and Meta-Analysis. Eur. J. Clin. Microbiol. Infect. Dis. 33 (3), 347–358. doi: 10.1007/s10096-013-1962-1 23979729

