# Supplementary material for: Development of Human Cell-Based In Vitro Infection Models to Determine the Intracellular Survival of Mycobacterium avium
Source: Front Cell Infect Microbiol. 2022 Jun 24;12:872361. doi: 10.3389/fcimb.2022.872361 (PMC9263196; doi:10.3389/fcimb.2022.872361)
Supplement: Supplementary file 1 [file DataSheet_1.docx]

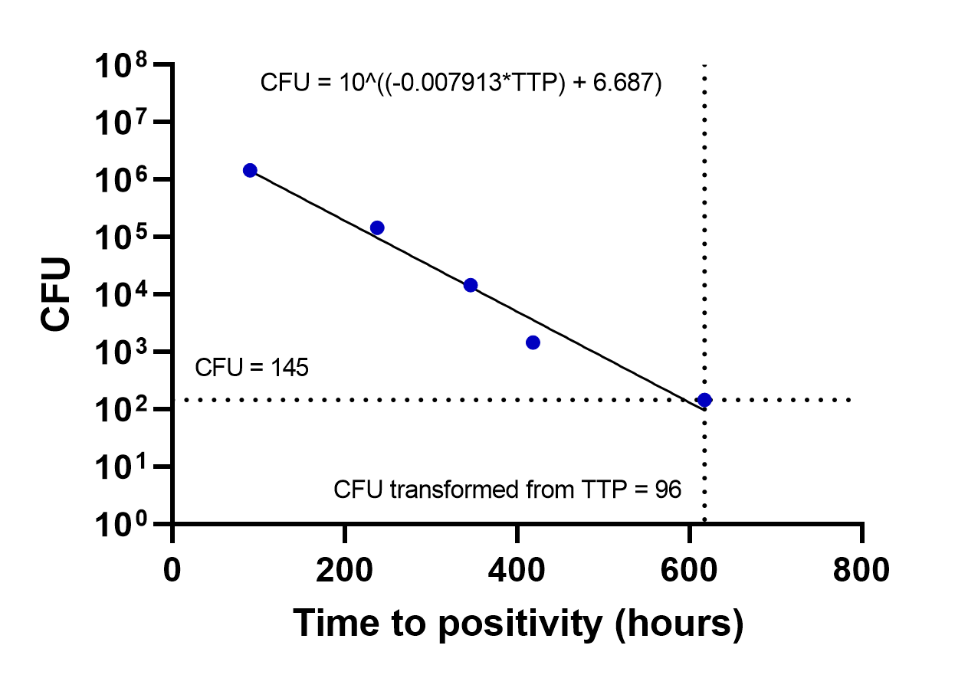


**Supplementary Figure 1. Example of relationship between plate-counted CFU and TTP values, with equation used to convert TTP values into CFU numbers.** For each MGIT experiment, the inoculum was serially diluted and CFU counts were determined by both the MGIT system and agar-plate counting. The plate-counted CFU and TTP measurements obtained for each dilution were plotted and linear regressed. The dotted lines represent the limit of detection for enumeration by classical CFU assay and the MGIT system. The equation derived from the linear regression was used to calculate CFU numbers from TTP values. ​


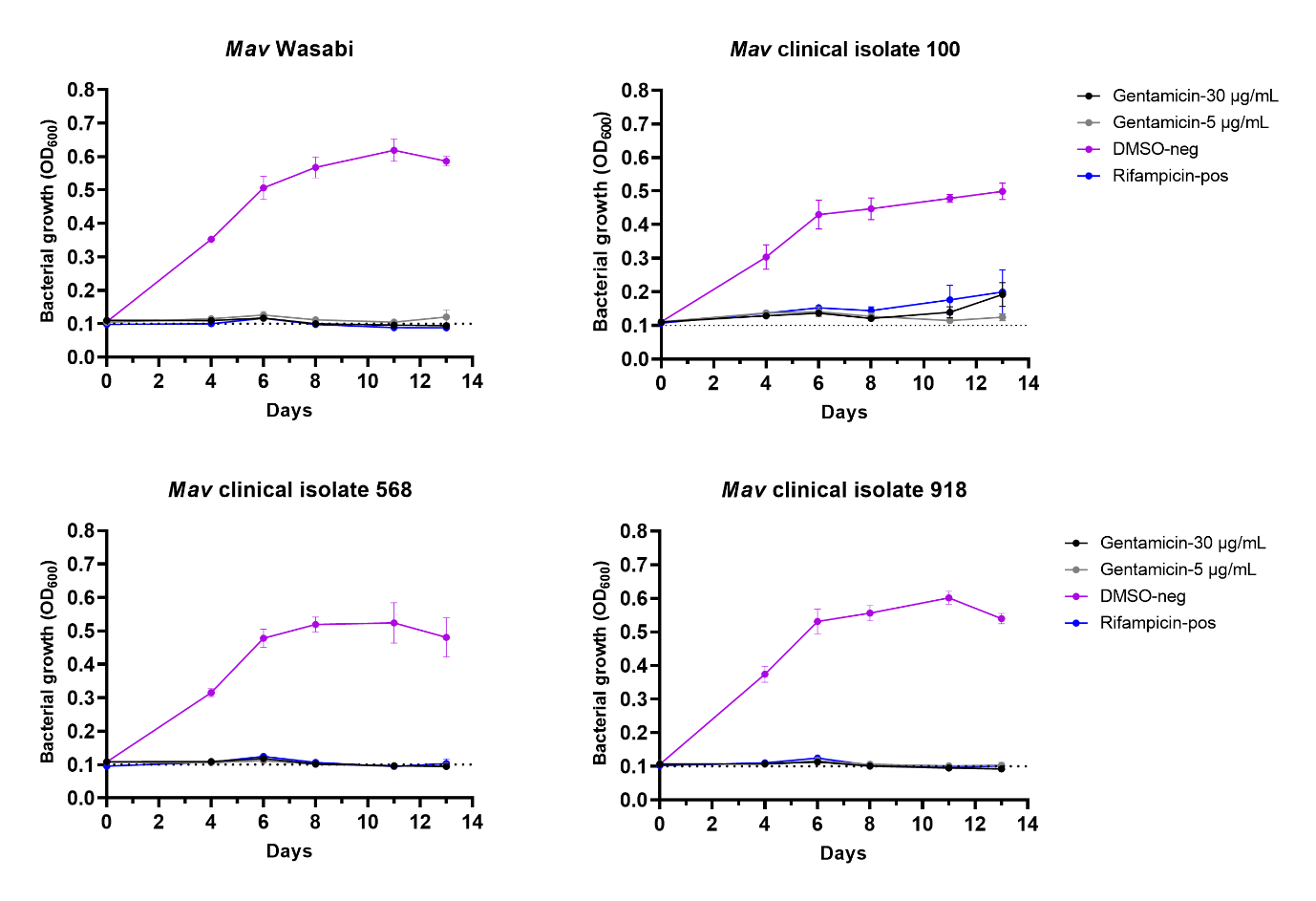


**Supplementary Figure 2. Susceptibility of *Mav* strains to gentamicin and the validity for gentamicin-use to kill extracellular bacteria in infection protocols.**  Susceptibility to gentamicin, which was used to kill extracellular bacteria in infection protocols, was determined for the four *Mav* strains. Liquid cultures of *Mav* were exposed to 5 μg/mL or 30 μg/mL gentamicin, DMSO (negative control) or rifampicin (positive control) and bacterial growth was monitored by absorbance measurements at 600 nm. Symbols and error bars represent the mean±SEM (n=4). ​


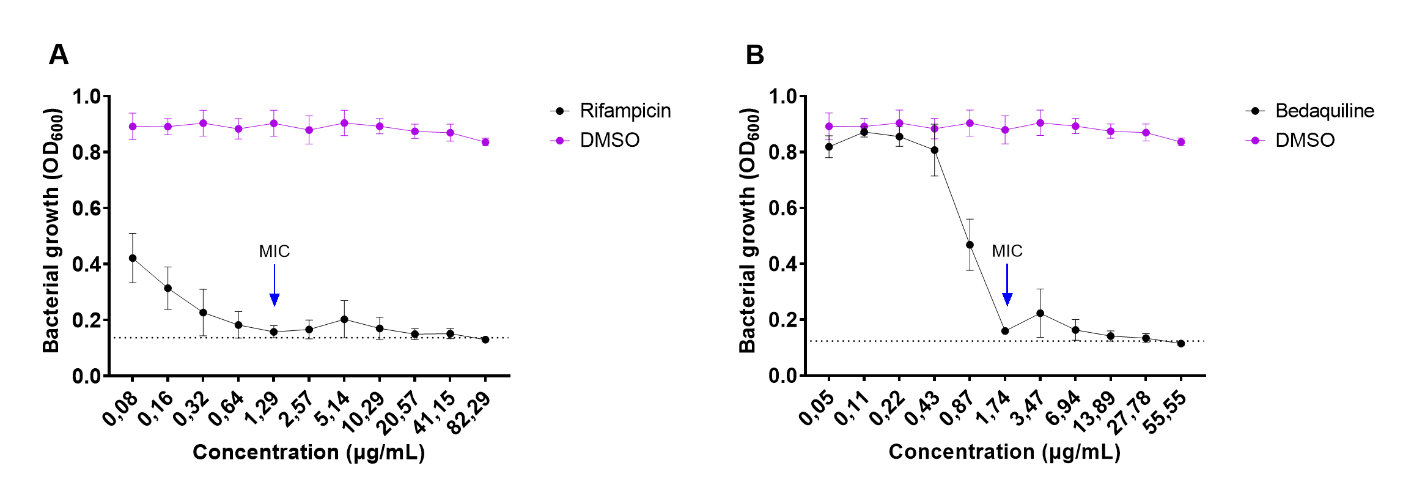


**Supplementary Figure 3. Determination of the MIC of rifampicin and bedaquiline for *Mav* Wasabi using the broth microdilution method.** The minimal inhibitory concentration (MIC) of rifampicin and bedaquiline was determined for *Mav* Wasabi, by exposing the bacteria in liquid broth to two-fold serial dilution of the antibiotics or control (DMSO). Bacterial growth was monitored by absorbance measurements at 600 nm. The arrow indicates the determined MIC. Symbols and error bars represent the mean±SEM (n=2).
